# Factors predicting health services use among older people in China: An analysis of the China Health and Retirement Longitudinal Study 2013

**DOI:** 10.1186/s12913-016-1307-8

**Published:** 2016-02-18

**Authors:** Cathy Honge Gong, Hal Kendig, Xiaojun He

**Affiliations:** Centre for Research on Ageing, Health and Wellbeing and ARC Centre of Excellence in Population Ageing Research, Australian National University, Canberra, Australia; Economics and Management Research Centre, Hunan University, Changsha, China; Centre for Research on Ageing, Health and Wellbeing, Australian National University, Florey, Building 54, Mills Road, Canberra, ACT 2601 Australia

**Keywords:** Healthcare utilization, Andersen model, Multivariate analysis, Socio-economic status, medical conditions, Healthy lifestyle, Health insurance, Older Chinese

## Abstract

**Background:**

Rapid population ageing in China is increasing the numbers of older people who are likely to require health services in response to higher levels of poor perceived health and chronic diseases. Understanding factors influencing health services use at late life will help to plan for increasing needs for health care, reducing inequalities in health services use and releasing severe pressures on a highly variable health care system that has constrained public resources and increasing reliance on health insurance and user payments.

**Methods:**

Drawing on the nationally representative China Health and Retirement Longitudinal Study 2013 data, we apply the Andersen healthcare utilization conceptual model to binary logistic regression multivariate analyses to examine the joint predictors of physical examinations, outpatient and inpatient care among the middle-aged and elderly in China.

**Results:**

The multivariate analyses find that both physical examinations and inpatient care rates increase significantly by age when health deteriorates. Females are less likely to use inpatient care. Significant socio-economic variations exist in healthcare utilization. Older people with higher education, communist party membership, urban residence, non-agricultural household registration, better financial situation are more likely to have physical examinations or inpatient care. Factors influencing all three types of health care utilization are household expenditure, losing a partner, having multiple chronic diseases or perceiving poor health. With activities of daily living limitations or pain increases the probability of seeing a doctor while with functional loss increases the rates of having physical examinations, but being the ethnic minorities, no social health insurance, with depression, fair or poor memory could be a barrier to having physical examinations or seeing a doctor, which might delay the early diagnose of severe health problems among these groups. Not drinking, not smoking and regular physical exercises are adaptations after having health problems.

**Conclusions:**

As a rapidly ageing society, in order to address the increasing needs and inequalities in health care utilization, China is facing a massive challenge to reform the current health care system, improve equitable access to health insurance and financial affordability for the most disadvantaged, as well as to provide more health education and information to the general public.

## Background

The use of health services in low-income country settings has been found to be influenced by health insurance, chronic diseases, age, gender, marital status, education, standard of living, and urban residence [1]. Factors found in related studies include socio-economic status measured by income and/or education) [2–5], ethnicity [6], health insurance [7], perceived health and chronic conditions [3, 4, 8], out of pocket costs [5, 9], regional health care supply [10] and transportation to health facilities [11]. Both the demand for and inequality in health care utilization can be expected to increase dramatically in China due to its rapid ageing population, changing health profiles and massive reforms including privatization in the health care system. To inform further service developments, there is an emerging need to better understand the key factors influencing health services use in China.

As a result of both the one-child policy introduced at the end of the 1970s and subsequent increases in life expectancies at older ages, China is becoming one of the most rapidly ageing countries in the world, growing older at a rate far faster than that in western developed countries, such as the US, UK and Australia. The population aged 60 years and over in China is projected to increase from 193 million (13.9 %) in 2013 to 454 million (32.8 %) by 2050, with a proportion of older people exceeded at that date only by Japan [12]. This trend could not be modified in the coming two or three decades by the recently announced cancellation of the one child policy.

Along with population ageing, China is experiencing significant health transitions with older people living longer generally in good health but also with increasing years in suboptimal perceived health accompanied by chronic diseases [13]. Globally and domestically, the population health challenges are transitioning from communicable, maternal, and neonatal health to an increasing focus on age-related non-communicable chronic diseases and disabilities [14–16].

Population ageing and changing health profiles are combining with rapid developments and reforms in the health care system in China resulting in an increase of inequalities in access to health care services [17]. China has gained significant improvements in both life and health expectancies in the last two to three decades due to the developments in living standard and medical treatment [13, 18]. However, there are great concerns about increases in medical costs, unnecessary admissions and overuse of drugs and equipment, as well as the deterioration of preventive programs in poor areas and growing gaps in health services use among sub population groups [17, 19, 20]. During the economic reforms, there has been a shift from the ideal of “equal access for equal need” to a profit-oriented hospital system that has constrained public resources and increasing reliance on health insurance and user payments [17, 19, 20]. For instance, poor people have had difficulty in accessing health services due to increasing medical costs and low insurance reimbursement rates; their poverty could be exacerbated once they become ill [17]. The New Cooperative Medical Scheme introduced in 2003 in the rural areas has improved access to health services for low income rural residents [21], financially protected rural households with members having chronic diseases [16] and distributed health benefits more equally [7]. Nonetheless, there still are significant gaps in access to services and problems with reimbursement rates and out of pocket costs among people having different health insurance schemes [9].

Several studies have to date investigated factors influencing health care utilization in China drawing on 2008 pilot study of China Health and Retirement Longitudinal Study (CHARLS) in Gansu and Zhejiang provinces [9, 17, 22, 23]. The main findings are:

(1) significant inequalities in use of both outpatient and inpatient care associated with different levels of income, access to health insurance schemes, availability of medical facilities [17], and levels of out-of-pocket payments [9]; (2) very low reimbursement rates [22], and underutilization of outpatient care among rural residents with the New Cooperative Medical Scheme. This is probably due to the moral hazard of the beneficiaries which could be induced by the insurance designed as a catastrophic insurance at household level [23]. Other studies report significant economic and distance barriers to use of health services by residents of a poor town in the Dabie Mountains [11], and financial difficulties as the leading reason for non-use of health services among ‘empty-nest’ elderly people in Shang Dong [24].

These studies were based on provincial or regional data which did not provide a national picture. To the best of our knowledge, our study provides the first available national study reporting findings from multivariate logistic regression analyses on factors jointly influencing healthcare utilization among people aged 45 years and over in China using the most recently published CHARLS 2013 data.

The Chinese health care system differs significantly from those in western countries. For instance, China has a hospital-centred health care system which is similar to the one in western countries at the beginning of twentieth Century. In China, people do not have access to general practitioners in private practice. Primary health care is provided by either a hospital or a community /village clinic for both emergency and non-emergency treatments. Referrals are not required although insurance might restrict the services use within local health care facilities. The application of the widely used Andersen health care utilization conceptual model [25–27] to the Chinese case will add important evidence to the accumulating international literature on how predisposing, enabling, and need factors influence health services use.

This article is organized as follows. The next section outlines the research methods including the data source, conceptual and statistical modelling approaches and key variables appropriate to the Chinese case. Findings are then presented firstly describing how each single factor is associated with health care utilization; and secondly by the multivariate regression model identifying factors jointly predicting physical examinations, visiting doctors and inpatient care. The discussion interprets the findings within the Chinese cultural and international context. The conclusion interprets the findings, study limitations and policy implications, as well as suggests directions for future research.

## Methods

### Data

This study is based on the nationally representative China Health and Retirement Longitudinal Study (CHARLS) 2013 survey conducted by the China Centre for Economic Research of Peking University. Face-to-face interviews in respondents’ homes collected detailed information on their demographic characteristics, socio-economic status, health-related behaviours and lifestyles, health status including health conditions, health insurance and health services use. In the first wave of CHARLS in 2011, older people aged 45 years or above living in China were randomly sampled using a multi-stage probability-proportional-to-size technique, stratified by regions and then by urban districts or rural counties and per capita gross domestic product. In the second wave in 2013, a total of 18246 respondents aged 45 and plus were followed up and surveyed. Individual weights in the CHARLS data, denoting the inverse of the probability that the observation is included because of the sampling design and the adjustment with household and individual non-response, were used for the analyses. More details on the CHARLS survey design are available from Zhao et al. [28].

### Andersen healthcare utilization conceptual model

The Andersen healthcare utilization model or behavioral model, developed in 1968 in the USA context, has been applied widely in international and US research on health services [25-27]. It has guided systematic investigations into the factors that lead to the use of health services (such as inpatient care, physician visits, dental care etc.) including predisposing, enabling and need factors. Predisposing factors can be characteristics such as age, sex, race, education, occupation, ethnicity, social relationship, social norms and health beliefs etc. Enabling factors could be income, wealth, access to health insurance, availability of transportation and health care facilities etc. Need represents both perceived and actual need for health care services, such as perception of health, evaluated health, injury and death rates etc. [25–27].

In understanding healthcare utilization in China, it is important to take account of additional factors, such as urban and rural residence and the household registration system which limit healthcare supply and mobility across regions. In addition, China has diverse ethnic groups with the dominant Han culture and a range of minorities. Socio-economic status is influenced by membership of the Communist party, occupation and income, as well as the central allocation of housing that can influence the availability of transportation and health care services [17, 29]. The majority of older Chinese are married and main care responsibilities rest primarily with spouses and younger family members many of whom are co-residents. Healthy lifestyles can be indicators of positive health beliefs and health literacy as influences on access to health care.

### Econometric model: binary logistic regression

Binary logistic regression models are used. Healthcare utilization is used as the dependent variable in the regression model which is measured by (1) physical examinations in the last 18 months; (2) visiting a doctor in the last month during illness; and (3) using inpatient care in the last year if required by a doctor. Physical examinations are indicators of preventive health actions. Visiting a doctor (including doctor home visit) is outpatient care, excluding any admission to medical facilities. The term “doctors” in China includes medically trained specialists, general practitioners and ‘bare-foot’ doctors in village clinics. It excludes nurses as they do not provide independent health consultations directly to patients. Inpatient care is defined as admission to a medical facility for overnight stay.

The predisposing factors in the model include age, gender, education, marital status, Communist party membership, ethnic group, household registration system and healthy life styles. Age has been divided into four ten-year groups: 45–54, 55–64, 65–74 and 75+. Education has three categories: (1) under primary school, including illiterate or semi-illiterate, under primary or home study; (2) schools without a degree, including middle and high schools as well as vocational education; and (3) college and above degrees, including college, bachelor, master and doctor degrees. Four marital statuses are used: (1) married with spouse present, including cohabitated; (2) married with spouse away for job purpose; (3) separated, divorced or widowed; and (4) single or never married. The second group “married with spouse away” is used as a separated group because some Chinese couples are living apart in different areas in order to meet their job commitment. Communist party membership might indicate some advantages in access to better economic resources and health care services as it is the only dominant party in China. Ethnicity refers to the 56 nationalities of which Han is the majority and the others are the minorities, such as Miao, Zhuang and Zhang etc. Whether having a local and what type of household registration system (agricultural or non-agricultural one) are major barriers in finding a formal job and accessing to social securities and health insurance schemes in an area. Healthy lifestyles, which serve as a proxy for health beliefs, are measured by three variables: (1) current, former or never a smoker; (2) drinking alcohol more than once or less than once in a month or never; (3) having regular physical exercises defined as doing exercises as at least 3 days per week and more than 30 min per day, including moderate to vigorous physical activities and walking.

The enabling factors in the model include urban or rural residence, relative living standard, household expenditure and health insurance. Urban and rural residents have different access to transportations and medical facilities. The urban and rural areas are defined according to the most recently published statistical standard by the National Bureau of Statistics, where urban areas include both communities and villages located within the access to the city or town’s facilities, while rural areas include villages only out of the city or town’s facilities. In order to better discern the impact of large variations across regions in both income and medical costs, we use both household annual expenditure and relative living standard to measure socio-economic status. The relative living standard is self-reported by answering the question “Compared to the average living standard of people in your city or county, how would you rate your standard of living, much better, a little better, about the same, a little worse, much worse?” Health insurance is measured by having any form of health insurance as below: (1) government medical insurance; (2) urban employee basic medical insurance; (3) urban resident basic medical insurance; (4) new cooperative medical scheme in rural area; (5) private commercial medical insurance purchased by respondents’ employers or by themselves; (6) other health insurance, including urban and rural resident medical insurance, medical aid, health insurance for severe illness etc.; or (7) no medical insurance.

The need factors in the model include self-reported health, chronic diseases, physical disabilities, functional loss, fair or poor memory, activities of daily living (ADL) limitations, depression and pain. Self-reported health is based on the respondents’ answer to the question “Would you say your health is excellent, very good, good, fair and poor?” or “Would you say your health is very good, good, fair, poor and very poor?” We have combined the answers of these two questions into one scale “excellent, very good, good, fair, poor and very poor”. Chronic diseases are assessed as the cumulative number of diagnosed conditions (hypertension, dyslipidaemia, diabetes, cancer, chronic lung diseases, liver or gallbladder disease, heart disease, stroke, kidney disease, stomach or other digestive disease, emotional, nervous, or psychiatric problems, memory-related disease, rheumatism/arthritis, asthma) on a scale from 0 to 3 and plus. Physical disabilities are self-reported disabilities from the response to the question “Do you have one of the following disabilities, physical disabilities?” Functional loss indicates any of the self-reported brain damage, mental retardation, vision problem (blind or semi-blind), hearing problem (deaf or semi-deaf) and speech impediment (dump or semi-dump). ADL limitations indicate any self-reported difficulty in any of the following activities of daily living domains as cut-off: bathing/showering, eating, dressing, getting into or out of bed, using toilet, or controlling urination and defecation. Depression is measured by a group of ten questions selected from the Center for Epidemiologic Studies Depression Scale on whether being bothered by things, having trouble concentrating on things, feeling depressed, feeling everything was an effort, not feeling hopeful about future, feeling fearful, having restless sleep, not being happy, feeling lonely or could not get going. A point of zero to three was assigned to each answer and then summed up and divided into three groups according to the distribution of total score: no, mild/moderate or major depression. Pain was queried by the question “Yesterday, did you feel any pain (no pain, a little pain, some pain, quite a lot pain and a lot pain)?”

## Results

### Sample characteristics

This study is based on a sample of 18246 completed respondents aged 45 years and over. Among the study population, about 33 % are aged 45–54 years, 35 % aged 55–64, 21 % aged 65–74 and 10 % aged 75 and plus. There are slightly fewer males than females. About one tenth are Communist party members, more than 90 % are Han ethnic group, one fifth have non-agricultural household registration and 40 % are living in urban areas. There is a certain proportion (about 20 %) of older people with agricultural household registration living in the urban areas, including residents in villages in urban areas and rural migrants in urban communities. Education levels are relatively low among older Chinese, with less than 3 % having college or above degrees, and slightly less than half being illiterate or semi-illiterate. The majority (79 %) are married and with spouse present, 6 % married with spouse away, 14 % divorced, separated or widowed and 1 % never married. The main care responsibilities rest primarily with spouses and younger family members many of whom are co-residents. Among older people who have reported whether or not they have physical exercises, only less than half (48 %) of older people report having regular physical exercises. One fifth are current smokers and 10 % former smokers; 26 % drink alcohol more than once per month, 8 % drink less than once per month and 66 % do not drink.

Among those who have compared their living standard to the average living standard of people in their cities or counties, about two-thirds (68 %) rate their living standard as relatively lower, less than one third about same and less than 5 % higher. The majority of older people (89 %) are covered by the three basic medical insurance schemes (11.7 % by urban employee basic medical insurance, 5.2 % by urban resident basic medical insurance and 72.0 % by new cooperative medical scheme. Another 7 % by government, private commercial or other types of health insurance, and still 4 % without any medical insurance.

In terms of health status, only one fourth report good or above health, half with fair health and another fourth with poor or very poor health; 3 % have reported physical disabilities and 9 % live with at least one ADL limitation; and around one tenth have had at least one severe functional loss in hearing, vision, speech or brain. The majority (82 %) have experienced fair or poor memory and about one tenth report mild/moderate depression and 3 % major depression. More than one fourth report a little to some level of pain and one tenth report quite a bit or a lot of pain. In terms of health services use, 37 % have had physical examinations in the last 18 months; 31 % have been sick in the last month of whom 22 % have seen a doctor; 17 % have been recommended to use inpatient care by a doctor in the last year but only 12 % have actually used inpatient care.

Health status is associated significantly with age. Comparing the youngest age group (aged 45 to 54) to the oldest age group (aged 75 plus), the proportion reporting good health decreases from 28 to 20 %, while the proportions increase from 19 to 31 % for reporting poor health and from 2 to 5 % for having very poor health. The proportions increase over these two age groups from 56 to 72 % for having at least one disease, from 11 to 24 % for having three or more diseases, from 2 to 5 % for having any physical disability, from 6 to 22 % for having any functional loss, from about 4 to 23 % for having any ADL limitation, from 11 to 12 % for having mild to major depression, from 14 to 20 % for having some to a lot of pain. However, we found healthier lifestyles at older ages. The proportion of former smokers has increased from 7 % for the youngest older people to 15 % for the oldest group and from 61 to 77 % for not drinking.

When people were asked why they did not see a doctor in the last month if they were ill, more than half of people (61 %) have reported no need, 12 % having self-medication, another 12 % with no money and 2 % for traffic reason. When asked why they did not use inpatient care in the last year if recommended by a doctor, more than half of people (55 %) have reported no money, 20 % were not willing, 1.5 % no ward available and 2 % for poor hospital quality.

### Descriptive analysis of single factor impact

Table 1 presents the three types of healthcare utilization by each predisposing, enabling and need factor (or by demographics, healthy lifestyles, socio-economic status, health insurance and health conditions). It is found that healthcare utilization differs significantly by age and other predisposing factors. For instance, inpatient care rates increase by age; people aged 65 and plus have higher proportions with physical examinations and doctor visits, which is consistent with age related health deterioration. There are no major gender differences in physical examinations and inpatient care, although females are much more likely to report having poor health and seeing a doctor. Well educated older people with college or above degrees are much more likely to have physical examinations and report good health while they are less likely to have inpatient care and see a doctor. Communist party members are more likely to have physical examinations and inpatient care although they have reported a higher proportion with good health and no significant difference in seeing a doctor. Married couple are more likely than single people to report good health; older people who are divorced, separated, widowed are more likely to have physical examinations, inpatient and outpatient care; while married people with spouse away and single people are less likely to have physical examinations and inpatient care. These patterns reflect both age effects and the importance of partnership in maintaining and improving health and health service use among older Chinese. The Han ethnic group have a higher probability than the ethnic minorities to be using all three types of health care. Residents in urban areas or those with non-agricultural household registration report higher proportions of having physical examinations, inpatient care and good health than their counterparts living in rural areas or with agricultural household registration.

**Table 1:** Healthcare utilization and perceived good health by predisposing, enabling and need factors among older Chinese, 2013

Health services utilization and perceived health	Physical examinations in the last 18 months	Inpatient care in the last year	Seeing a doctor in the last month	Good perceived health
Total	36.7	11.9	21.5	23.8
Age 45–54	30.9	8.9	19.4	27.9
Age 55–64	33.7	11.0	20.6	22.9
Age 65–74	47.3	14.7	25.0	20.5
Age 75+	44.8	18.5	24.0	20.3
Male	36.5	12.4	19.0	26.5
Female	37.1	11.7	23.7	21.5
Under primary school	33.2	12.7	23.0	20.7
School without degree	38.4	11.6	20.3	25.9
College and above degrees	73.5	11.2	17.4	44.3
Communist party member	53.2	16.1	21.8	28.9
Not Communist party member	34.7	11.4	21.4	23.2
Married with spouse presented	36.8	11.8	20.7	24.8
Married with spouse away	28.2	9.1	19.5	22.3
Separated/divorced/widowed	40.7	15.0	26.4	20.7
Single	30.7	7.0	23.4	11.7
Han	37.5	12.1	21.7	24.1
Minority	28.7	11.6	17.7	22.2
Agricultural household registration	31.8	11.6	21.3	23.0
Non-agricultural household registration	53.0	13.3	21.5	26.9
Other household registration	50.5	7.9	19.6	32.0
Rural resident	31.1	11.3	21.5	22.0
Urban resident	45.0	13.2	21.2	26.7
Better living standard	51.1	12.1	19.7	42.9
About same living standard	42.3	11.7	20.5	28.6
Worse living standard	34.0	11.6	22.3	19.6
Average or above household expenditure	44.3	14.3	22.7	25.6
Lower than average household expenditure	33.2	11.0	20.8	23.2
Government insurance	63.8	16.2	22.4	31.5
Urban employee insurance	60.7	14.5	24.0	28.0
Urban resident insurance	42.6	12.1	20.6	25.0
New rural cooperative insurance	31.9	11.8	21.4	22.1
Commercial insurance	49.1	9.4	16.0	38.9
Other type of insurance	45.6	12.7	23.2	26.9
No insurance	25.5	9.1	15.8	28.8
No chronic disease	30.6	7.7	14.6	35.2
One chronic disease	35.5	12.0	22.1	21.4
Two chronic diseases	39.2	14.3	24.4	13.8
Three and more chronic diseases	47.5	20.0	32.5	10.0
Diseases not reported	37.3	10.5	19.5	30.0
Any physical disability	38.5	16.9	26.7	10.3
No physical disability	36.8	11.9	21.3	24.8
Any functional loss	41.4	18.0	27.8	12.9
No functional loss	36.2	11.3	20.6	25.3
Good perceived health	36.2	6.2	9.9	100.0
Fair perceived health	36.2	9.6	19.3	
Poor perceived health	38.2	21.2	35.3	
Very poor perceived health	39.2	26.7	38.2	
With any ADL	41.8	25.4	32.5	5.8
Without ADL	36.9	13.3	24.8	16.7
ADL not reported	35.6	6.9	12.5	41.7
No depression	37.9	10.4	19.6	26.9
Moderate/mild depression	32.8	15.9	31.7	7.4
Major depression	30.1	18.7	39.7	3.6
Depression not reported	35.0	16.0	21.6	22.6
Fair/poor memory	36.3	11.8	23.0	18.6
Good memory	41.0	10.8	14.7	48.3
No pain	37.7	10.3	16.9	30.5
A little pain	36.5	13.5	27.8	14.9
Some pain	36.4	14.9	31.6	10.7
Quite a lot pain	34.1	16.1	33.8	5.1
A lot pain	35.0	15.6	36.5	5.5
Current a smoker	30.7	8.8	18.0	26.4
Former a smoker	43.7	21.4	21.3	19.6
Never a smoker	37.7	11.4	22.9	23.2
Drinking alcohol more than once per month	33.7	7.8	16.1	29.8
Drinking alcohol less than once per month	38.2	9.0	21.1	26.4
Not drinking alcohol	37.9	14.1	23.6	21.3
Regular physical exercises	38.2	12.1	23.3	22.3
Less than regular physical exercises	32.4	10.0	21.8	23.2
No physical exercise	33.6	18.9	18.4	19.1
Not reported	37.5	12.1	21.0	24.7

Data source: Authors’ own calculation using CHARLS 2013 national data

Surprisingly, older people in China with healthy lifestyles, such as not smoking or drinking,, with regular physical exercises, are having higher rates of reporting not good health and having physical examinations, inpatient and outpatient care when compared to their counterparts who are currently smoking, drinking or having less regular physical exercises. No physical exercise (less than ten minutes per day) is an indicator of poor perceived health and need for inpatient care. These findings differ from western developed countries where in general not smoking, not drinking and regular physical exercises are all related to better health outcomes hence less healthcare service use. The plausible explanation could be that the Chinese people might not fully recognize that not drinking, not smoking and regular physical exercises are healthy behaviours so they may prioritize these healthy behaviours and health care usage as adaptations after having health problems.”

Enabling factors are more important for physical examinations and inpatient care than for seeing a doctor. Older people with higher household expenditure are having higher rates of all three types of healthcare utilization and reporting good perceived health, while those with self-reported better relative living standard are more likely to have physical examinations and inpatient care though less likely to see a doctor. Certain health insurance schemes have better access to health services. For instance, older people with government medical insurance or urban employee basic medical insurance are more likely to have used all three types of health care although they report relatively good health. Older people with commercial insurance have reported higher proportions of good perceived health and having physical examinations but lower proportion for using inpatient care and seeing a doctor.

Medical conditions and functional limitations are all positively associated with healthcare utilization excepting that depression, pain or poor memory could be a motivational barrier for physical examinations. Among people with three or more chronic diseases, 48 % have had physical examinations, 20 % have used inpatient care, and 33 % have visited a doctor, while these proportion are 31, 8, and 15 % for those without any chronic disease. People with any physical disability, functional loss, ADL limitations or poor or very poor perceived health have higher probability in using the three types of healthcare utilization. People with mild/moderate or major depression, poor memory or pain are more likely to use inpatient care or visit doctors but less likely to have physical examinations.

### Multivariate regression analysis of joint impacts

We run the binary logistic regression models to examine the joint predictors of physical examinations, inpatient care and visiting a doctor by controlling all the predisposing, enabling and need factors. The estimated coefficients that describe the effects of the predictors are presented in Table 2. We have also checked the overall significance of the models by reporting the Wald chi-square value and P-value from the multivariate logistic regression models at the bottom of Table 2, as well as conducting the likelihood ratio test between two models (one with predisposing factors only and the other with all predisposing, enabling and need factors). This shows that the overall significance of the models is high and adding the enabling and need factors can significantly improve the model fitness.

**Table 2:** Joint predictors of health care utilization among older Chinese, 2013

Health services utilization	Physical examinations		Inpatient care		Visiting doctors	
	Coefficient	P > z	Coefficient	P > z	Coefficient	P > z
Age 45–54						
Age 55–64	0.137	0.023	0.096	0.322	−0.119	0.107
Age 65–74	0.691	0.000	0.199	0.050	0.048	0.570
Age 75+	0.733	0.000	0.405	0.003	−0.095	0.429
Male						
Female	0.102	0.165	−0.290	0.004	−0.048	0.578
Under primary school						
School without degree	0.142	0.006	−0.032	0.704	0.018	0.780
College and above degrees	0.886	0.000	−0.201	0.388	0.014	0.940
Communist party member	0.355	0.000	0.228	0.022	0.047	0.616
Not communist party member						
Married with spouse presented						
Married with spouse away	−0.239	0.108	−0.186	0.289	0.024	0.847
Separated/divorced/widowed	0.152	0.064	0.185	0.059	0.259	0.017
Single	0.460	0.079	−0.331	0.514	0.055	0.848
Han						
Minorities	−0.410	0.000	−0.132	0.253	−0.424	0.000
Agricultural household registration						
Non-agricultural household registration	0.220	0.019	0.015	0.923	−0.043	0.700
Other household registration	0.347	0.096	−0.298	0.380	−0.146	0.512
Urban resident	0.227	0.000	0.166	0.082	0.044	0.540
Rural resident						
Better living standard						
About same living standard	−0.216	0.083	0.026	0.887	−0.046	0.762
Worse living standard	−0.360	0.003	−0.084	0.643	−0.118	0.425
Not reported	−0.300	0.027	−0.123	0.538	−0.049	0.763
Average or above household expenditure	0.209	0.000	0.294	0.000	0.139	0.000
Lower than average household expenditure						
Government insurance						
Urban employee insurance	0.275	0.147	−0.077	0.743	−0.011	0.960
Urban resident insurance	−0.297	0.172	−0.232	0.414	−0.336	0.171
New rural cooperative insurance	−0.244	0.216	−0.097	0.707	−0.299	0.171
Commercial insurance	0.170	0.452	−0.151	0.632	−0.472	0.071
Other types of insurance	0.149	0.523	−0.029	0.920	−0.077	0.785
No medical insurance	−0.557	0.029	−0.236	0.588	−0.673	0.008
No chronic disease						
One chronic disease	0.201	0.004	0.267	0.026	0.279	0.001
Two chronic diseases	0.316	0.000	0.367	0.001	0.284	0.001
Three and more chronic diseases	0.568	0.000	0.488	0.000	0.462	0.000
Disease not reported	0.183	0.061	0.124	0.368	0.149	0.141
Any physical disability	0.135	0.269	0.039	0.818	−0.051	0.695
Not physical disability						
Any functional loss	0.166	0.017	0.131	0.142	0.060	0.419
No functional loss						
Good perceived health						
Fair perceived health	0.045	0.453	0.469	0.000	0.593	0.000
Poor perceived health	0.183	0.012	1.262	0.000	1.202	0.000
Very poor perceived health	0.224	0.108	1.435	0.000	1.213	0.000
With ADL	0.043	0.697	0.130	0.291	0.188	0.079
Without ADL						
Not reported	0.077	0.197	−0.176	0.111	−0.312	0.000
No depression						
Moderate/mild depression	−0.180	0.018	0.177	0.065	0.137	0.069
Major depression	−0.280	0.030	0.241	0.138	0.313	0.014
Not reported	−0.089	0.299	0.157	0.190	0.012	0.902
Fair/poor memory	0.091	0.147	0.1262	0.166	−0.1982	0.010
Good memory						
No pain						
A little pain	−0.044	0.501	0.033	0.762	0.360	0.000
Some pain	−0.008	0.924	0.076	0.486	0.448	0.000
Quite a lot pain	−0.099	0.276	−0.051	0.671	0.371	0.000
A lot pain	−0.051	0.677	−0.197	0.249	0.444	0.001
Not reported	−0.031	0.957	−1.551	0.055	−0.255	0.670
Current a smoker						
Former a smoker	0.484	0.000	0.979	0.000	−0.036	0.797
Never a smoker	0.330	0.000	0.403	0.001	0.171	0.117
Not reported	0.240	0.006	0.455	0.000	0.044	0.683
Drinking alcohol more than once per month						
Drinking alcohol less than once per month	0.023	0.804	0.022	0.870	0.201	0.103
Not drinking alcohol	0.107	0.095	0.513	0.000	0.218	0.004
Regular exercises						
Less than regular exercises	−0.071	0.360	−0.149	0.225	−0.061	0.523
No exercise	−0.134	0.253	0.301	0.054	−0.542	0.000
Not reported	0.032	0.603	−0.094	0.349	−0.109	0.160
Wald chi2(52)	816.22		669.64		943.31	
Prob > chi2	0.0000		0.0000		0.0000	

Data source: Authors’ own calculation using CHARLS 2013 national data

The estimated efficient is significant when its *P*-value is small than 0.10

With the exception of insignificance of having any physical disability, all other factors have significant impacts on health care utilization, but factors associated with higher probabilities of inpatient care differ from those for physical examinations; factors associated with higher probability of visiting a doctor are very different from those predicting physical examinations or inpatient care. For instance, after controlling for other factors, older people aged 65 plus, are more likely to have had physical examinations and inpatient care, but age becomes insignificant for visiting doctors.

The impacts of the joint predictors from the multivariate regression models could be summarized as:

1. Being female is negatively correlated to inpatient care, possibly reflecting the fact that females are more capable to manage their health problems in a better way than males;
2. higher education is positively associated with physical examinations probably because highly educated older people are more attentive to their health;
3. Communist party membership and urban residence are positively associated with both physical examinations and inpatient care mainly because Communist party members and urban residents have better access to medical facilities and better forms of health insurance;
4. non-agricultural household registration is positively associated with physical examinations while being the ethnic minorities is negatively associated with both physical examinations and visiting a doctor;
5. being separated/divorced/widowed is positively associated with all three types of healthcare utilization indicating that partnership is playing an important role in maintaining good health among older people;
6. household expenditure is reported as the most important enabling factor for all three types of health care utilization while self-reported relative living standard is only significant for having physical examinations;
7. no social health insurance (no medical insurance or with private commercial insurance) is negatively associated with physical examinations or seeing a doctor; the benefits of government or urban employee medical insurance schemes become insignificant once household expenditure is controlled;
8. multiple chronic diseases and poor perceived health are strong need factors for all three types of healthcare utilization; depression increases the probability of using inpatient care and visiting a doctor, with ADL limitations or pain increases the probability of seeing a doctor while with functional loss increases the probability of having physical examinations, but with depression, fair or poor memory could be a barrier to having physical examinations or seeing a doctor, which might delay the early diagnose of severe health problems among these groups;
9. currently not drinking, or not smoking is positively associated with having physical examinations and using inpatient care, while the difference between having regular or less than regular physical exercises becomes insignificant. This may reflect adaptive behaviours after having health problems instead of reduced health risk factors as have been found in the literature on developed countries.

## Discussion

Our results show that the oldest age groups are having more physical examinations and inpatient care in line with their higher proportions reporting poor perceived health, chronic diseases, disabilities, ADL limitations, functional loss, pain and depression, as well as less regular physical exercises. The proportion of older people who currently do not smoke or drink increases with age, possibly attributable in part to selection and cohort effects, and in part to reflect a healthier life style which appears when older people pay more attention to their health with advancing age and deteriorating health.

There are strong socio-economic variations in healthcare utilization. “No money” is the main reported reason for a significant proportion of older people for not using inpatient care even if required by a doctor although lack of money is only a partial reason for not seeing a doctor when ill. Self-medication is an option for primary health care when medical costs are high or access to health care services is limited [30]. Health status for older adults of ethnic minorities, or those living in remote rural areas, is mostly unfavourable as their access to health care services is often limited. Conversely, Communist party members, urban residents with formal employment, and highly educated people are relatively advanced.

Household expenditure is the strongest enabling factor for three types of healthcare utilization, while self-rated relative living standard matters only for physical examinations. Certain types of health insurance, such as government medical insurance and urban employee medical insurance, have more benefits than others in enabling better access to healthcare, while their impacts become insignificant after controlling for household expenditure. The plausible explanation could be that older people with government medical insurance and urban employee medical insurance are more likely to have higher household expenditure. No social health insurance (no medical insurance or with private commercial insurance) is negatively associated with physical examinations or seeing a doctor

Multiple chronic diseases and perceived poor health are the main need factors influencing all three types of healthcare utilization; poor memory is negatively while having ADL limitations or pain is positively associated with seeing doctors; with functional loss increases the rates while with depression could be a psychological barrier to accessing physical examinations.

Our findings are in general consistent with the international ones [1–4, 6–8, 10] although there are some specific findings reflecting Chinese cultural and policy context which are different from those in Western developed countries. For instance, healthy life styles, such as not drinking or not smoking, are pronounced adaptive healthy behaviours of older Chinese after having health problems. Communist party members and urban residents with formal jobs are advanced in accessing to medical facilities and they have better forms of health insurance. Conversely, the minorities and those living in remote rual areas are most vulnerable in health services use. Older people with depression are less likely to have preventative health examinations and those with poor memory are less likely to see a doctor which might delay the diagnosis of any severe health problem.

When compared to the previous studies in China, similar conclusions are drawn that socio-economic status, financial capacity, chronic diseases, perceived poor health and health care provision are the most important factors influencing health service use among older Chinese [17]. However, our study applying rigorous multivariate regression analyses to the 2013 national data extended the findings. For instance, we found that socio-economic status as measured by household expenditure has a significant impact on all three types of healthcare utilization while the self-reported relative living standard measure only has a significant influence on physical examinations. Further, we show that females have lower rates of inpatient care than males, and rural seniors have relatively lower rates of all three types of healthcare utilization. These findings are different from those of the previous study [31] showing that both rural seniors and females have lower hospital utilization rates, but higher rates of physician visits, than their male and urban counterparts.

## Conclusion

This study is the first comprehensive application of the Andersen healthcare utilization conceptual model and rigorous multivariate analyses to the national data of CHARLS 2013. It has examined the joint factors predicting healthcare utilization among older Chinese, including physical examinations, outpatient care and inpatient care.

The findings from the multivariate regression indicate that both physical examinations and inpatient care rates increase significantly by age when health deteriorates but age is not significant for seeing a doctor. Females are less likely to use inpatient care although they have reported higher rates of poor perceived health. Significant socio-economic variations exist in healthcare utilization. Older people with higher education, communist party membership, urban residence, non-agricultural household registration, higher household expenditure or relatively better living standard are more likely to have physical examinations or inpatient care. Factors influencing all three types of health care utilization are financial capacity measured by household expenditure, losing a partner, having multiple chronic diseases or perceiving poor health. With ADL limitations or pain increases the probability of seeing a doctor while with functional loss increases the rates of having physical examinations, but being the ethnic minorities, no social health insurance, with depression, fair or poor memory could be a barrier to having physical examinations or seeing a doctor, which might delay the early diagnose of severe health problems among these groups. Not drinking nor smoking are found to be adaptive behaviours after having health problems.

The findings in this study will deepen our understanding of factors jointly predicting health services use in an ageing society of a middle income country. The results can inform actions to direct scarce resources to those in greatest needs taking into account the available supply in different areas and affordability issue for health services use. The findings have implications for actions to promote healthy and productive ageing as well as to improve quality of life when the proportions of older people increase rapidly in China.

The ageing population in China will increase needs for the use of current health care system that has severe shortages of supply. The challenges are heightened by not only increasing health care cost and growing numbers of older people but also by the rising prevalence of complex health problems including chronic diseases, functional loss, depression and pain notwithstanding the large numbers who remain active and without disability. As others have reported, the capacity of the health system in China to reliably provide primary care for identification and treatment of chronic diseases is limited by inadequate funding and unevenly distributed facilities, as well as an insufficient skilled workforce [14, 17, 18, 32].

Reforms responsive to increasing variability and inequalities in health services use could include regulating and financially encouraging cost effective prescribing and care performance, establishing more explicit diagnostic criteria, and better patient supervision to avoid inappropriate admissions and drug use in the health care system [20, 33]. On the supply side, more funding and innovation funding could be directed to poor areas and improving the primary health infrastructures in rural area [33]. As was the case with developments in the West from the 1960s, primary health care needs to be strengthened with better identification and treatment of chronic diseases; and moving more service provision from hospitals to more cost efficient community care centres (or a family doctor-led primary care model) with necessary training support and relocation of staff and resources. These structural improvements are essential to improve the efficiency and equity in health services use in order to decrease the severe use and cost pressures on hospitals in an ageing society [34–36]. Difficulties in accessing health services by the ethnic minorities living in remote rural areas could also be improved by providing more community health provision and interventions [6].

Health care needs and inequalities can be addressed through enhancing demand capacities by providing universal or more equal access to health insurance and more financial assistance to disadvantaged groups [24]. This can include reducing deductibles and coinsurance rates or increasing reimbursement rates for rural residents covered by the new cooperative medical scheme in poor areas; providing financial assistance to those on low incomes who cannot afford health insurance; and adequately addressing the special needs of the aging population with chronic health conditions [8].

More fundamentally, individual health can be improved over the life course through health education and information provided to the general population, especially to low income groups, through official media or community health workers [37]. Healthy lifestyles – notably not smoking nor drinking and adequate physical activities – are important. Our results indicate older persons’ health beliefs that good health can be maintained better through healthier behaviours and preventative physical examinations as well as more medical consultations in the community relative to inpatient care.

CHARLS is an excellent longitudinal survey on China but ours should be interpreted with some caution as there are certain limitations which should be noted. Firstly, we have only used the cross sectional associations to examine the predictors for healthcare utilization without aiming to understand the underlying causality. Secondly, CHARLS is a representative community sample in urban and rural areas but it does not cover the about 1 % older people living in an institution [16, 28]. Thirdly, CHARLS uses self-report measures of relative living standard, health status and functional limitations which are more prone to measurement error than clinical or performance assessments. These measurement issues are particularly important considerations for those in rural areas with limited access to health professionals and health information and hence low awareness of health diagnosis criteria [14, 18].

Future research could further explore the effects of structural features on both the supply and demand sides of healthcare utilization in China. It will be important to take account of segmentation and constraints, for example, in terms of urban and rural residence, socio-economic status, health insurance schemes and out of pocket payments. Another priority will be to examine the longitudinal changes for individuals as they age, as well as changes in China’s health and health services performance over time. This needs to take into account the impacts of services developments along with individual ageing, cohort succession and broader social changes. Similarities and differences identified in health services utilisation between China and Western countries, as per this article, indicates the potential value of cross-national comparisons for understanding the influence of different cultural, policy and socio-economic contexts of ageing.

## Appendix 1

**Table 3:** Summary of characteristics of study population, 2013

Variables	Values	Frequency	Unweighted %	Weighted %
Total	Aged 45 +	18246	100	100
Age group	Aged 45–54	5920	32.5	33.4
	Aged 55–64	6694	36.8	35.2
	Aged 65–74	3736	20.5	20.5
	Aged 75+	1855	10.2	10.2
Gender	Male	8807	48.3	48.4
	Female	9428	51.7	51.6
Education	Under primary school	8182	44.9	44.9
	School without degree	9633	52.8	52.9
	College and above degrees	414	2.3	2.2
Communist party member	Yes	1987	10.9	10.7
	No	16212	89.1	89.3
Marital status	Married with spouse present	14764	81.0	78.7
	Married with spouse away	1035	5.7	6.2
	Separated/Divorced/Widowed	2273	12.5	14.2
	Single	155	0.9	1.0
Ethic group	Han	16787	92.4	92.4
	Minorities	1374	7.6	7.6
Residence	Rural	10881	59.7	58.7
	Urban	7361	40.4	41.3
Household registration	Agricultural	13893	76.4	76.6
	Non-agricultural	4090	22.5	22.3
	Other	211	1.2	1.2
Relative living standard	Better	503	3.9	3.6
	About same	3595	27.7	28.1
	Worse	8891	68.5	68.4
	Not reported	5253		
Health insurance	Government	253	1.4	1.3
	Urban employee	2150	12.0	11.7
	Urban resident	904	5.0	5.2
	New rural cooperative	12939	72.2	72.0
	Commercial	393	2.2	2.2
	Other types	555	3.1	3.2
	No medical insurance	730	4.1	4.4
Physical examinations in the last 18 months	Yes	6444	36.7	36.8
	No	11118	63.3	63.2
Seeing a doctor in the last month when ill	Yes	3773	21.0	21.4
	No	1700	9.3	9.2
	Not applicable	12502	69.6	69.4
Inpatient care in the last year if necessary	Yes	2134	11.8	12.1
	No	900	5.0	4.7
	Not applicable	15042	83.2	83.3
Number of diseases	No chronic disease	4846	32.6	33.7
	One chronic disease	4430	29.8	29.9
	Two chronic diseases	2844	19.2	18.6
	Three and more chronic diseases	2728	18.4	17.8
	Not reported	3394		
Any physical disability	Yes	539	3.1	3.0
	No	16908	96.9	97.0
Any functional loss	Yes	2047	11.3	10.9
	No	16030	88.7	89.1
Perceived health	Good and above	4276	23.7	23.9
	Fair	8863	49.1	49.2
	Poor	4358	24.2	23.8
	Very poor	550	3.1	3.0
Any ADL limitation	Yes	1114	8.9	9.4
	No	11360	91.1	90.6
	Not reported	5621		
Depression	No	13087	86.6	87.1
	Mild/Moderate	1585	10.5	10.1
	Major	443	2.9	2.8
	Not reported	2980		
Fair/poor memory	Yes	13520	83.1	82.4
	No	2751	16.9	17.6
Pain	None	10545	64.3	65.2
	A little	2915	17.8	17.6
	Some	1383	8.4	8.3
	Quite a bit	1051	6.4	6.1
	A lot	519	3.2	2.9
	Not reported	1829		
Smokers	Current a smoker	2938	20.1	20.3
	Former a smoker	1355	9.3	9.5
	Never a smoker	10296	70.6	70.3
	Not reported	3653		
Drinking alcohol	More than once a month	4794	26.6	26.2
	Less than once a month	1441	8.0	7.9
	No	11780	65.4	65.9
Physical exercises	Regular exercises	2855	48.0	48.1
	Less than regular exercises	2484	41.8	41.8
	No exercise	611	10.3	10.2
	Not reported	12145		

Data source: Authors’ own calculation using CHARLS 2013 national data

## Appendix 2

**Table 4:** Health status and medical conditions by age group (weighted %), 2013

	Aged 45–54	Aged 55–64	Aged 65–74	Aged 75+	Total
Good perceived health	27.9	22.9	20.5	20.3	23.8
Fair perceived health	51.3	50.6	46.9	43.9	49.3
Poor perceived health	18.8	23.6	29.0	30.8	23.9
Very poor perceived health	2.0	2.8	3.7	5.1	3.0
No chronic disease	44.4	33.0	23.8	28.0	33.7
One chronic disease	29.2	31.0	29.0	29.5	29.9
Two chronic diseases	15.3	18.6	22.8	18.9	18.6
Three and more chronic diseases	11.1	17.3	24.4	23.7	17.8
Any physical disability	2.1	2.8	3.7	5.0	3.0
Any functional loss	5.9	9.5	15.1	22.3	10.9
Any ADL limitation	3.5	6.9	11.0	23.4	9.5
No depression	88.9	86.0	85.4	88.2	87.1
Moderate/mild depression	8.9	10.8	11.3	9.9	10.2
Major depression	2.2	3.3	3.2	1.9	2.8
Fair/poor memory	80.5	84.4	83.3	81.3	82.6
No pain	68.0	64.3	62.9	62.6	65.1
A little pain	18.2	17.2	17.6	17.3	17.6
Some pain	7.4	8.4	8.9	9.9	8.3
Quite a lot pain	4.5	6.6	7.0	7.5	6.1
A lot pain	1.9	3.6	3.6	2.7	2.9
Current a smoker	22.3	20.7	19.8	12.2	20.1
Former a smoker	6.6	9.2	11.9	15.4	9.5
Never a smoker	71.1	70.1	68.3	72.4	70.4
Drinking alcohol more than once per month	29.3	27.2	24.6	17.7	26.3
Drinking alcohol less than once per month	9.4	8.0	6.4	5.7	7.9
Not drinking alcohol	61.4	64.8	69.1	76.7	65.9
Regular physical exercises	50.1	46.5	50.5	41.3	48.1
Less than regular physical exercises	43.5	44.9	37.4	32.8	41.8
No physical exercise	6.4	8.7	12.1	25.9	10.2

Data source: Authors’ own calculation using CHARLS 2013 national data

## Abbreviations

CHARLS: China Health and Retirement Longitudinal Study
ADL: activities of daily living
